# Beyond the brain: Polyglutamine disease pathology outside the nervous system

**DOI:** 10.1016/j.nbd.2026.107539

**Published:** 2026-07-18

**Authors:** Julia P. Rausch, Brock Bradley, Fabio Demontis, Sokol V. Todi

**Affiliations:** a Department of Pharmacology, Wayne State University School of Medicine, Detroit, MI 48201, USA; b Department of Developmental Neurobiology, St. Jude Children's Research Hospital, Memphis, TN 38105, USA; c Department of Neurology, Wayne State University School of Medicine, Detroit, MI 48201, USA

**Keywords:** Adipose tissue, Aging, Ataxia, Atrophy, Blood, Bone, CAG repeat expansion, Cardiovascular system, Endocrine system, Gastrointestinal system, Glia, Gonadal system, Immune system, Liver, Muscle, Neuron, Skin

## Abstract

Polyglutamine diseases are primarily age-dependent neurodegenerative disorders, but patient-based data also point to a broader, multisystem biology. Clinical, imaging, biochemical, and post-mortem studies support peripheral involvement across multiple organ systems in the onset and progression of Huntington's Disease, Spinal and Bulbar Muscular Atrophy, Dentatorubral-Pallidoluysian Atrophy, and six Spinocerebellar Ataxias. Various peripheral abnormalities often emerge before or alongside overt neurological symptoms, contribute to disability and mortality, and are only partly explained by deconditioning or medications. Current data support viewing polyglutamine diseases as systemic protein-misfolding syndromes with organ-selective vulnerability, where peripheral tissues both mirror and modify CNS pathology in humans. Here, we propose that integrated, longitudinal, multisystem phenotyping and targeted organ-directed interventions are essential components of future research investigations, clinical care, and trial design.

## 1. Introduction

**Fig. 1.**
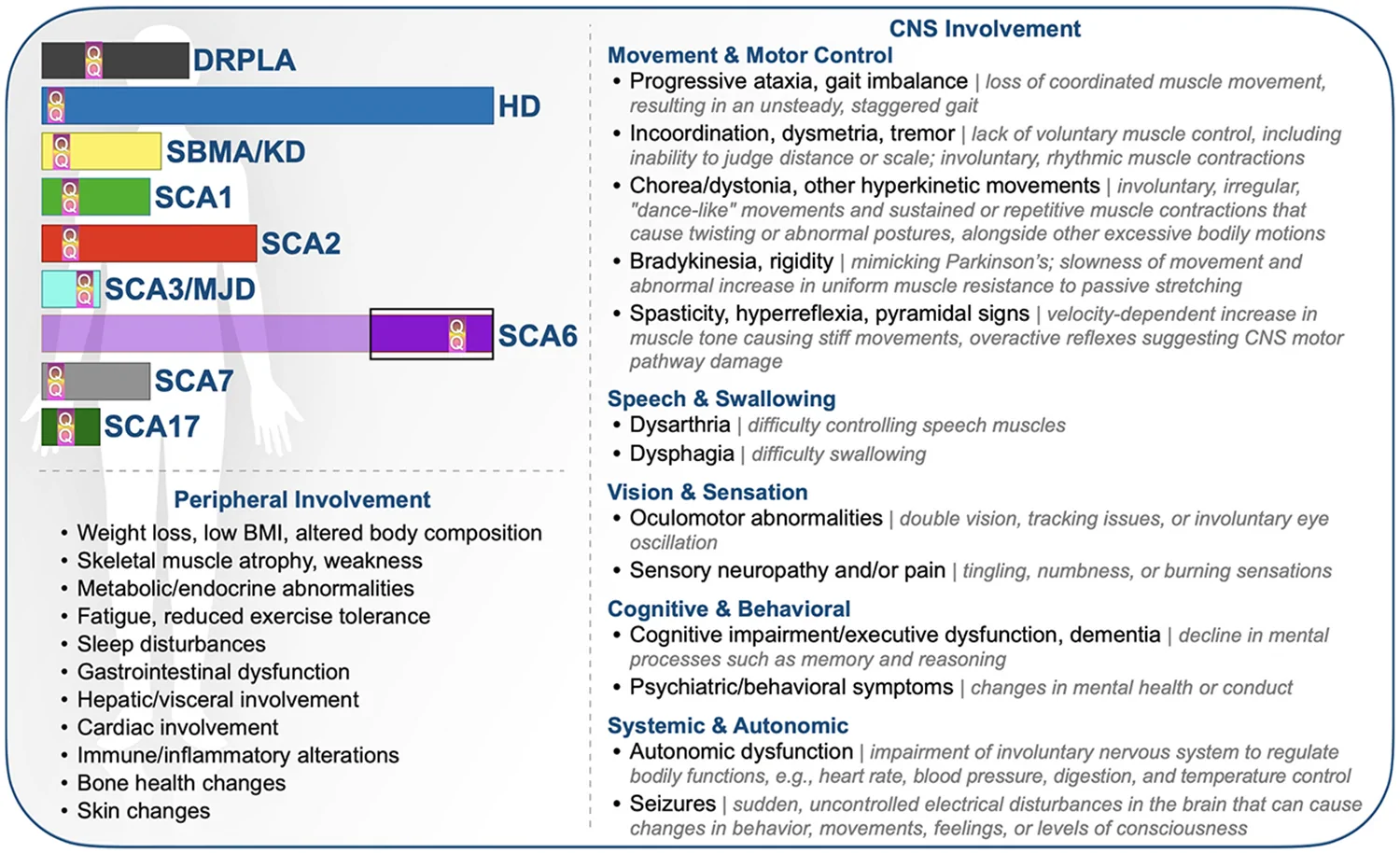
The family of polyglutamine diseases and observed symptoms. Box in SCA6 outlines the bicistronic portion of the gene that leads to the translation of the segment of *CACNA1A* that is thought to most directly cause this disease. Abbreviations for the diseases are in the main text and in Table 2.

Polyglutamine (polyQ) diseases are a group of age-dependent, inherited neurodegenerative disorders caused by CAG repeat expansions encoding abnormally long glutamine tracts in otherwise unrelated proteins. This family includes Huntington's disease (HD; caused by mutations in the protein Huntingtin), spinal and bulbar muscular atrophy (SBMA, also known as Kennedy's disease; androgen receptor), dentatorubral–pallidoluysian atrophy (DRPLA; atrophin-1), and six spinocerebellar ataxias (SCAs 1, SCA2, SCA3/Machado–Joseph disease, SCA6, SCA7, and SCA17; respectively ataxins 1, 2, 3, CACNA1A, a bicistronic gene that encodes the α1A P/Q-type calcium-channel subunit and the C-terminal transcription factor α1ACT, ataxin-7, and TATA-binding protein; Fig. 1 and Table 1). The relevant proteins are widely or ubiquitously expressed, yet studies in general have emphasized selective central nervous system (CNS) vulnerability, particularly of the striatum in HD and cerebellum/brainstem in SCAs (reviewed in Todi et al., 2007; Paulson et al., 2017; Lieberman et al., 2019; Johnson et al., 2022).

Clinically and genetically, the canonical polyQ diseases share several unifying features (Table 1). All are slowly progressive neurodegenerative conditions in which age at onset is, in broad terms, inversely correlated with CAG repeat length, and most are fully penetrant above a disease-specific threshold. HD is the most common polyQ disease in Western populations and presents with a characteristic triad of movement disorder (typically chorea), psychiatric symptoms, and cognitive decline, reflecting predominant degeneration of striatal and cortical projection neurons (The Huntington's Disease Collaborative Research Group, 1993; Ghosh and Tabrizi, 2018). In contrast, SBMA is X-linked and essentially restricted to males; it is characterized by slowly progressive lower motor neuron degeneration with bulbar and limb weakness, often accompanied by endocrine manifestations of partial androgen insensitivity, such as gynecomastia and reduced fertility (La Spada et al., 1991; La Spada and Taylor, 2003). DRPLA, relatively frequent in Japan but rare elsewhere, combines features of ataxia, chorea, myoclonus, seizures, and cognitive decline, with juvenile-onset cases dominated by epilepsy and intellectual deterioration, whereas adult-onset cases resemble a hybrid of HD and SCA presentations (Naito and Oyanagi, 1982; Koide et al., 1994; Nucifora Jr et al., 2003). SCA1, SCA2, and SCA3 are paradigmatic autosomal dominant ataxias. They share early gait ataxia, dysarthria, and progressive limb incoordination, with neuropathology centered on cerebellum, brainstem, and connected pathways. SCA1 and SCA2 primarily affect Purkinje cells and brainstem nuclei, whereas SCA3 shows broader involvement including cerebellar nuclei, substantia nigra, globus pallidus, and anterior horn cells, accounting for its mixed ataxia–pyramidal–extrapyramidal phenotype (Banfi and Zoghbi, 1994; Kawaguchi et al., 1994; Stevanin et al., 1995; Nucifora Jr et al., 2003). SCA6 is clinically and genetically distinct: it is usually a late-onset, “pure” cerebellar ataxia caused by a relatively small CAG expansion in the *CACNA1A* gene and progresses more slowly than other SCAs (Sasaki et al., 1998; Zhuchenko et al., 1997; Lieberman et al., 2019; Du et al., 2013; Johnson et al., 2022). SCA7 combines cerebellar ataxia with progressive cone–rod dystrophy, making it unique among polyQ diseases for its prominent retinal degeneration and its particularly dramatic anticipation with severely large paternal expansions causing infantile-onset multisystem disease (David et al., 1997; Helmlinger et al., 2004). SCA17 typically presents with a mixture of ataxia, cognitive decline, and extrapyramidal features; in some cases, it can closely mimic HD clinically, reflecting widespread cerebral involvement (Zühlke et al., 2001; Toyoshima et al., 2005; Todi et al., 2007; Lieberman et al., 2019). Within SCAs, impairment of eye movement and other ocular abnormalities are relatively frequent symptoms. SCA2 patients can present with thinner retinal structures and higher cup-to-disc ratios (Rezende Filho et al., 2024), and, as already stated above, SCA7 patients can show macular deterioration, reduced corneal endothelial cell density, and a high prevalence of visual impairment (Campos-Romo et al., 2018). A cross-sectional study in SCA3 found esodeviation at long distances and in lateral gaze (Lemos et al., 2021).

Despite this clinical diversity, the polyQ diseases share a common molecular feature: in each, the expanded polyQ tract alters the folding of the host protein and promotes misfolding, oligomerization, and aggregation, eliciting a primarily toxic gain-of-function that is modulated by the surrounding protein context, such as domains, interaction partners, subcellular localization signals, and post-translational modifications, as well as by partial loss of the protein's normal functions (Muchowski and Wacker, 2005; Todi et al., 2007; Lieberman et al., 2019; Johnson et al., 2022). Protein quality control pathways, including the ubiquitin-proteasome system and autophagy, are recruited to manage aggregation-prone species, but can become overwhelmed or functionally altered. Nuclear localization of mutant proteins or toxic fragments, transcriptional dysregulation, mitochondrial dysfunction, calcium dyshomeostasis, and disrupted axonal transport have all been implicated to varying degrees across the family, with the precise balance of mechanisms differing by disease protein and cell type (Todi et al., 2007; Paulson et al., 2017; Lieberman et al., 2019; Johnson et al., 2022).

**Fig. 2.**
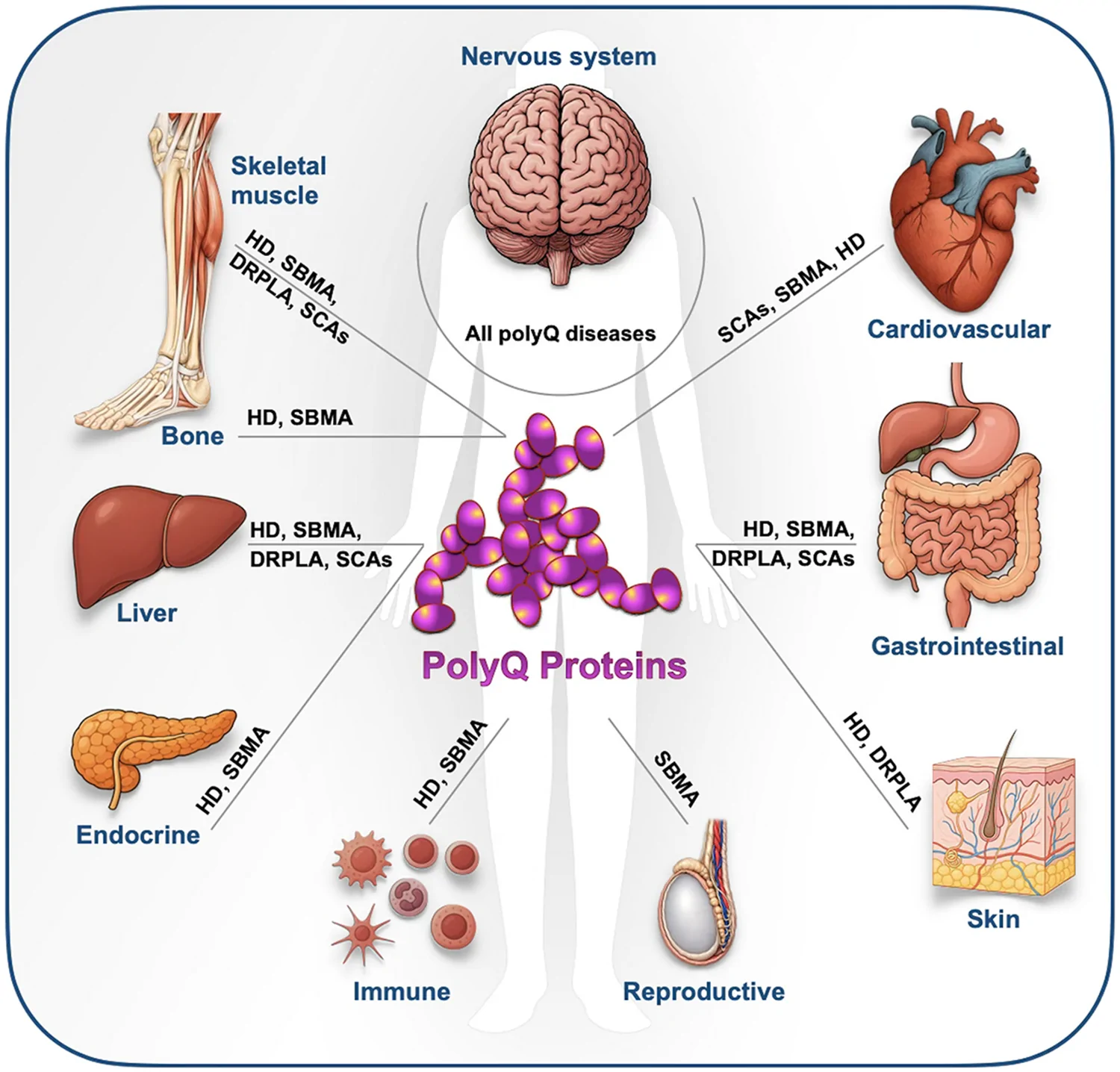
Summary of peripheral effects of polyQ diseases. Abbreviations are the “Introduction” in the main text. The cartoon representation of polyQ proteins is not data-based.

Over the past three decades, it has become clear that the concept of polyQ diseases as purely CNS disorders is incomplete (Fig. 2). Patient-based studies using muscle biopsies, in vivo phosphorus-31 magnetic resonance spectroscopy (31P-MRS; abbreviations are listed in Table 2), dual-energy X-ray absorptiometry (DEXA), endocrine profiling, liver imaging and biopsy, autonomic testing, skin histology, and blood-based biomarkers have revealed broad involvement of skeletal muscle, liver, endocrine organs, bone, adipose tissue, skin, the cardiovascular system, and the immune system in individuals carrying polyQ expansions (Table 3). These findings are often detectable in premanifest or early-stage patients, arguing for intrinsic peripheral pathology rather than late-stage consequences of disability or malnutrition.

Here, we summarize patient-based evidence for non-neural organ involvement across the polyQ disease family. We focus on data derived directly from mutation carriers, clinical cohorts, case-control and longitudinal studies, case series, and reports involving imaging, laboratory measures, and tissue biopsies; we refer to animal and cell-based work only when helpful to contextualize human findings. By organizing the data by organ system and disease, we highlight shared and disease-specific patterns and consider how these systemic manifestations may influence prognosis, symptom burden, and ongoing and future therapeutic strategies. Table 3 summarizes most of the information that is presented in the following sections.

## 2. Skeletal muscle involvement

Skeletal muscle has traditionally been viewed as a secondary casualty in polyQ diseases. However, converging patient-based data indicate that HD, SBMA, DRPLA, and multiple SCAs all feature intrinsic muscle abnormalities. In the following sections, we summarize skeletal muscle involvement across these disorders: HD and SBMA are relatively well characterized, DRPLA remains sparsely documented, and the polyQ SCAs are beginning to be explored more widely.

### 2.1. Muscle bioenergetic deficiency in HD

Clinically, HD patients frequently report fatigue, muscle wasting, and weakness that cannot be fully explained by central motor features. For example, in a clinical study Busse and colleagues assessed isometric strength across six lower-limb muscle groups using hand-held dynamometry in 20 symptomatic HD participants and 20 age- and sex-matched controls (Busse et al., 2008). HD patients exhibited clear weakness (on average ~ 50% of control strength); composite strength indices were consistent with clinically meaningful peripheral contributors to reduced mobility and endurance (Busse et al., 2008).

Early evidence for intrinsic skeletal muscle pathology in HD came from in vivo studies (Lodi et al., 2000; Saft et al., 2005; Ciammola et al., 2006, 2011). One investigation examined the calf muscle in 12 HD patients (4 of whom were premanifest) and 2 DRPLA patients, together with healthy controls, using 31P-MRS of the calf at rest and after standardized exercise (Lodi et al., 2000). Symptomatic HD and DRPLA subjects exhibited a reduced resting phosphocreatine (PCr)/inorganic phosphate (Pi) ratio and a significantly delayed post-exercise PCr recovery half-time. The investigators also observed an estimated ~35–44% reduction in maximal mitochondrial ATP production in symptomatic and premanifest HD, and a similar deficit in DRPLA, consistent with impaired oxidative phosphorylation in vivo (Lodi et al., 2000). The presence of abnormalities in presymptomatic carriers supports an intrinsic disease-related component and argues against deconditioning as the sole explanation.

Subsequent 31P-MRS work reinforced the conclusions from the study above by probing skeletal muscle oxidative metabolism in both manifest HD and asymptomatic mutation carriers (Saft et al., 2005). The authors found delayed post-exercise phosphocreatine recovery kinetics, indicating impaired mitochondrial oxidative phosphorylation capacity. The fact that this anomaly was also observed in mutation carriers (Saft et al., 2005) supports the idea that muscle bioenergetic/mitochondrial dysfunction can occur before overt neurological disability is fully established and may contribute to fatigability and reduced exercise tolerance. A contemporaneous report that utilized cultured, primary skeletal muscle cells established from HD patients supported this conclusion and provided further mechanistic information by observing increased susceptibility to apoptosis, the presence of Huntingtin (HTT)-positive inclusions, and abnormalities in differentiation toward mature myotubes (Ciammola et al., 2006).

Additional evidence linking mutant HTT-dependent effects in skeletal muscle bioenergetics came from Turner and colleagues, who found that skeletal muscle mitochondrial respiratory-chain (complex II/III) activity correlated with clinical disease progression and cognitive impairment in HD (Turner et al., 2007), and also from Ciammola and colleagues, who evaluated metabolic responses during exercise in HD. The latter study reported reduced anaerobic threshold with increased skeletal muscle lactate production during exertion, consistent with an early shift toward anaerobic metabolism and/or constrained oxidative capacity (Ciammola et al., 2011). Together with structural and biochemical evidence of mitochondrial abnormalities described in related work, these data align with the view that muscle bioenergetics is perturbed in HD.

Body composition studies provided a complementary perspective. An initial investigation used DEXA in a study of 25 premanifest HD gene carriers and matched controls. Premanifest individuals had significantly lower bone mineral density z-scores despite similar body mass index (BMI) and largely normal testosterone, cortisol, leptin, and vitamin D levels, indicating an early, gene-related skeletal and muscular phenotype (Goodman and Barker, 2011). These findings were extended to patients with manifest HD by a later investigation, which observed that both lean mass and fat distribution were altered in clinically affected individuals, which were proposed as potential indicators of weight-loss risk (Costa de Miranda et al., 2019).

Further refinement of the emerging picture of HD-related pathology outside of the nervous system came from quantitative studies that reported reduced maximal voluntary contraction and decreased cross-sectional volumetric area of key muscle groups, even when controlling for age and activity level (Zielonka et al., 2014). Needle-biopsy studies in HD described altered mitochondrial network morphology and variable impairment of oxidative phosphorylation/respiratory control (Gehrig et al., 2017; Neueder et al., 2024); however, in premanifest HD mutation carriers, high-resolution respirometry indicated normal mitochondrial respiratory function (Buck et al., 2017). Lastly, a single-case report described biopsy-supported myopathy or myositis preceding the onset of overt chorea, highlighting that clinically relevant muscle disease can emerge early in HD (Kosinski et al., 2007). Collectively, spectroscopy, body-composition, strength, and biopsy data describe a potential scenario in which intrinsic, mitochondrial-driven myopathy and progressive sarcopenia may represent early and clinically meaningful components of the HD phenotype rather than late, secondary complications.

### 2.2. Motor impairment in SBMA

SBMA has long been recognized as a lower motor neuron disease with prominent muscle involvement. However, patient-based pathology suggests that muscle damage is not purely neurogenic. Muscle biopsies from SBMA patients often indicate a mixture of neurogenic and myopathic changes including fiber type grouping, fiber splitting, necrosis, and regeneration, indicative of primary myopathy superimposed on motor neuron degeneration (Querin et al., 2016, 2018). Creatine kinase (CK) levels are frequently elevated several-fold, and quantitative muscle testing reveals slowly progressive weakness over decades. Beyond the observation of classic neurogenic changes, muscle biopsies and ultrastructural analyses in a cohort of genetically confirmed SBMA patients and controls indicated increased mitophagy and abnormal mitochondrial morphology in skeletal muscle, together with denervation and fiber-type grouping (Borgia et al., 2017).

Large metabolic cohort studies further reinforce the concept that the muscle is a central peripheral target tissue in SBMA. One such investigation evaluated 55 genetically confirmed SBMA patients and 483 controls, systematically characterizing metabolic parameters and body composition. SBMA patients had lower BMI than controls and a high prevalence of insulin resistance that correlated with measures of motor impairment, although visceral fat area did not differ significantly between the groups (Nakatsuji et al., 2017). An investigation of 80 SBMA patients in a German cohort, assessing 28 biochemical markers of neuromuscular injury, metabolism, and endocrine function, identified elevations in CK and transaminases, as well as alterations in lipid profiles, further underscoring the systemic, muscle-centered nature of SBMA (Rosenbohm et al., 2018). A contemporaneous study also noted that muscle-derived, but not neuron-derived, biomarkers correlated best with clinical severity, reinforcing the primacy of muscle pathology in this disease (Lombardi et al., 2019).

Overall, convergent biopsy, metabolic, and biomarker data indicate that SBMA is not only a lower motor neuron disease, but also a systemic myopathic and metabolic disorder in which skeletal muscle serves as a primary peripheral target and major determinant of clinical severity.

### 2.3. Potential myopathy in DRPLA

Compared with HD and SBMA, patient-based data on skeletal muscle in DRPLA are sparse. The 31P-MRS study by Lodi et al. included two DRPLA patients, one premanifest, who underwent calf muscle spectroscopy alongside HD patients and controls; both individuals had reduced PCr/Pi ratios, delayed PCr recovery after exercise, and an estimated ~46% reduction in maximal mitochondrial ATP production, closely mirroring the muscle bioenergetic deficits seen in HD (Lodi et al., 2000).

An isolated case report described a DRPLA patient who underwent muscle biopsy for unexplained myopathy before genetic diagnosis. Biopsy of the rectus femoris of an individual who died of DRPLA at a very young age revealed lack of type IIB fibers, a predominance of type I fibers, a reduced average diameter across all fiber types, and increased lipid within type I fibers, suggesting a primary myopathic process (Cox et al., 2000). Adding to a developing view, early-onset DRPLA patients frequently develop hypoalbuminemia, sometimes triggered by febrile illnesses, and disease worsening is accompanied by increased urinary protein loss (Nagai et al., 2013). While not strictly a muscle phenotype, this systemic protein loss complicates nutritional status and may exacerbate muscle wasting, highlighting the interaction between systemic and neuromuscular pathology in DRPLA. These limited data suggest that clinically meaningful skeletal muscle involvement occurs in DRPLA, but its prevalence, natural history, and relationship to systemic manifestations remain undefined and require dedicated cohort studies.

### 2.4. Reduced mobility in SCAs

In SCAs, skeletal muscle has received less direct attention than in HD and SBMA. Patients often complain of fatigue, cramps, and subjective weakness (Todi et al., 2007; Paulson et al., 2017; Lieberman et al., 2019; Johnson et al., 2022; Leeuwenberg et al., 2026). However, reports of systematic muscle imaging, biopsy, or 31P-MRS in SCAs are not abundant. Nonetheless, the few case reports and small biopsy series that have been published suggest that some SCA genotypes may develop primary myopathic changes, including inclusion body myositis-like pathology and autophagic vacuolar myopathy, most often reported in SCA3 and SCA6 (Rietveld et al., 2020; Li et al., 2023). Chronic disability and reduced mobility in SCAs plausibly predispose patients to sarcopenia and secondary muscle changes. Prospective studies using DEXA, quantitative muscle ultrasound, and 31P-MRS in genetically defined SCAs are needed to delineate primary versus secondary muscle involvement.

As skeletal muscle is a major determinant of whole-body energy expenditure and glucose handling, the muscle phenotypes described above intersect closely with systemic metabolic regulation. Next, we consider endocrine and metabolic alterations across polyQ diseases.

## 3. Endocrine and metabolic systems

Systemic metabolic disruption is increasingly recognized as a core feature of several polyQ diseases. In this section, we summarize available evidence, which comes primarily from HD and SBMA.

### 3.1. Glucose homeostasis and diabetes in HD

HD patients exhibit a complex pattern of glucose dysregulation. A landmark cross-sectional study of 29 largely normoglycemic HD patients and 22 matched controls used glucose tolerance assessment, insulin sensitivity, and insulin secretion estimation to dissect glucose-insulin homeostasis (Lalić et al., 2008). The authors observed that HD patients had impaired insulin sensitivity, reduced peripheral glucose disposal, increased insulin resistance indices, and a marked reduction in early-phase insulin secretion despite normal fasting glucose (Lalić et al., 2008).

Clinical series have long noted an increased prevalence of diabetes in HD families. A survey of reported evidence concluded that diabetes can precede, accompany, or follow motor onset, with a cumulative prevalence higher than in the general population (Montojo et al., 2017). However, there is also evidence of normal glucose tolerance in HD cohorts: in a 24-h metabolic study of premanifest and stage II/III HD gene carriers, one study found no gross differences in standard carbohydrate markers between patients and controls (Nambron et al., 2016). Despite discrepancies, metabolomic and targeted metabolic profiling studies point to broader disturbances in energy metabolism. The latter report, for example, noticed altered profiles of amino acids, acylcarnitines, and other metabolites, consistent with impaired mitochondrial substrate handling (Nambron et al., 2016). A contemporaneous study identified distinctive plasma metabolic signatures in HD, including changes in amino acids and lipid metabolites that differentiate patients from controls (Cheng et al., 2016). Together with cachexia and weight loss, these findings support the idea that HD involves a systemic dysregulation of energy homeostasis in which glucose handling is only one component.

### 3.2. Endocrine and metabolic phenotype in SBMA

SBMA provides one of the clearest examples of endocrine disruption among polyQ diseases. A cross-sectional endocrine analysis of men with genetically confirmed SBMA and healthy controls documented a characteristic partial androgen insensitivity phenotype in SBMA, marked by gynecomastia, reduced fertility, elevated or high-normal serum testosterone, increased sex hormone-binding globulin, and inappropriately elevated gonadotropins, together with altered androgen receptor binding (Dejager et al., 2002). Additional metabolic cohorts reinforced the systemic reach of this phenotype. In one cross-sectional investigation, previously mentioned, SBMA patients were compared with age- and sex-matched healthy controls. They had lower BMI than controls, a high prevalence of insulin resistance, and higher insulin resistance that correlated with worse motor function (Nakatsuji et al., 2017). An Italian cohort of men evaluated by clinical examination, fasting laboratory testing, and liver ultrasound likewise reported that metabolic syndrome, insulin resistance, and non-alcoholic fatty liver disease were common in SBMA; among those who underwent abdominal sonography, non-alcoholic fatty liver disease was detected in 92% of the cases (Francini-Pesenti et al., 2018, 2020; additional information regarding liver disease in SBMA is found in section 4). Complementing these findings, another study highlighted how endocrine, skeletal, metabolic, hepatic, and cardiac features cluster into a broad non-neural disease burden (gynecomastia, testicular atrophy, osteoporosis, metabolic syndrome, non-alcoholic fatty liver disease, and cardiac conduction anomalies) that correlated with motor impairment (Querin et al., 2016). Additional investigations reported that clinical and biochemical indices of partial androgen insensitivity correlate with CAG repeat length, and that the endocrine phenotype is reproducible across case series and broader syntheses (Dejager et al., 2002; Valera Yepes et al., 2015). Collectively, these studies frame SBMA as a systemic disorder of androgen signaling and energy homeostasis, with practical implications for screening, risk stratification, and targeted management beyond motor neuron degeneration.

### 3.3. Endocrine disruptions in other polyQ diseases

Endocrine and metabolic data in DRPLA and SCAs are less extensive. Hypoalbuminemia in early-onset DRPLA may reflect albumin leakage from multiple organs and contributes to edema, malnutrition, and susceptibility to infections (Nagai et al., 2013). In SCAs, scattered reports describe metabolic and autonomic features, such as impaired insulin sensitivity/secretion in SCA1 and multiple observations of altered autonomic control by heart-rate variability and baroreflex metrics, yet systematic endocrine phenotyping comparable to what has been reported in HD or SBMA is limited (Lalić et al., 2010; Pradhan et al., 2008; Asahina et al., 2010; Montes-Brown et al., 2010; Montes-Brown et al., 2012; Tamuli et al., 2019).

These collective findings point to broader disturbances in nutrient handling and energy balance, with direct consequences for hepatic and gastrointestinal physiology. Next, we focus on liver and gastrointestinal involvement.

## 4. Hepatic and gastrointestinal involvement

Across several polyQ diseases, abnormalities ranging from subclinical metabolic liver dysfunction and non-alcoholic fatty liver disease (NAFLD) to overt protein-losing enteropathy and infantile multiorgan failure point to a broader, systemic disturbance of hepatic and GI homeostasis in polyQ patients. In this section, we summarize evidence from HD, SBMA, DRPLA, and the SCAs regarding liver involvement and hepatic risks as the diseases manifest through infancy and adulthood.

### 4.1. Liver involvement in HD

Although liver involvement in HD has been studied less extensively than muscle and endocrine dysfunction, the available literature indicates that hepatic metabolism is not spared. Surveys of metabolic and neuroendocrine features of HD reported that peripheral metabolic dysfunction is a component of disease biology, even if the liver has not been examined systematically compared to other peripheral tissues (Petersén and Björkqvist, 2006; Aziz et al., 2007; van der Burg et al., 2009; Chuang and Demontis, 2021). Patient-based studies support the view that HD is accompanied by broad metabolic abnormalities that are relevant to liver function, although the findings are heterogeneous across cohorts. Earlier work indicated altered glucose handling, impaired insulin sensitivity or secretion in some patients, changes in body fat stores, altered fatty-acid metabolism, and abnormalities in cholesterol biosynthesis and cholesterol-derived metabolites (Petersén and Björkqvist, 2006; Aziz et al., 2007; van der Burg et al., 2009; Nambron et al., 2016). But, more tightly controlled metabolic profiling has not consistently confirmed major abnormalities in fasting glucose, insulin, or lipid markers, indicating that peripheral metabolic alterations in HD are likely variable, stage-dependent, and sensitive to cohort composition and study design (Nambron et al., 2016). Thus, current human data support systemic metabolic dysregulation in HD, but they do not yet define a uniform clinical hepatic phenotype.

Several observations nevertheless point toward hepatic involvement. Brain-based clinical studies have reported elevated lactate levels in HD, consistent with impaired oxidative energy metabolism (Harms et al., 1997; Jenkins et al., 1993). In parallel, translational work has reported progressive hepatic mitochondrial dysfunction in both premanifest gene carriers and patients with early HD, suggesting that liver bioenergetic defects may emerge before advanced clinical disease (Hoffmann et al., 2014; Stüwe et al., 2013). These findings extend the argument beyond nonspecific weight loss or altered serum metabolites and support the idea that the liver may contribute actively to the systemic metabolic phenotype of HD.

Interest in anaplerotic therapy in HD emerged from this broader context of systemic energy deficit and early weight loss. A study reported decreased plasma branched-chain amino acids in HD, which were consistent with increased demand for Krebs cycle substrates (Mochel et al., 2007). These findings set the groundwork for testing triheptanoin, an odd-chain medium-chain triglyceride that replenishes Krebs cycle intermediates, as a potential corrective substance for metabolic anomalies in HD (Mochel et al., 2010). In a short-term pilot study of six patients, triheptanoin was well tolerated, increased propionylcarnitine and C5 ketone bodies, normalized end-exercise muscle acidosis in the two patients with pretreatment abnormalities, and significantly increased serum IGF-1, thereby providing proof of principle that anaplerotic therapy could improve peripheral energy metabolism in HD (Mochel et al., 2010). More recently, triheptanoin was evaluated in a phase II study in early-stage HD. The trial did not meet its primary endpoint, as triheptanoin did not reduce caudate atrophy over 6 months compared with placebo (Mochel et al., 2025). However, patients treated for 12 months showed motor stability, and post hoc comparison with an external placebo group suggested a reduction in the rate of caudate atrophy together with stabilization of motor scores. These findings are encouraging but should be interpreted cautiously because the 12-month benefit analyses were post hoc and the controlled primary endpoint at 6 months was negative.

Altogether, evidence supports a more assertive, but still cautious, conclusion that the liver is likely an active participant in the systemic pathophysiology of HD rather than a passive bystander. Future work should therefore move beyond general serum metabolic profiling and incorporate dedicated hepatic phenotyping in HD, including imaging, functional metabolic studies, and, where feasible, direct assessment of steatosis and hepatocellular dysfunction.

### 4.2. NAFLD and steatohepatitis in SBMA

In SBMA, liver involvement is well documented. One study examined two SBMA cohorts using a multi-modal cross-sectional approach that combined serum chemistries, ultrasound, MRI-based proton density fat fraction measurements, and liver biopsy in a subset (Guber et al., 2017). The investigators found that most SBMA patients exhibited hepatic steatosis on imaging, and biopsy specimens observed steatohepatitis with ballooning, inflammation, and varying degrees of fibrosis; hepatic fat content showed a trend toward correlation with CAG repeat length and metabolic indices (Guber et al., 2017). In the imaging cohort of this report, hepatic steatosis was detected in all 22 participants by at least one imaging modality; among those who underwent liver ultrasound (*n* = 16), steatosis was present in 12 (Guber et al., 2017). As also mentioned above, an independent study working with an Italian cohort of SBMA patients found evidence of non-alcoholic fatty liver disease in 22 out of 24 patients when using abdominal sonography (Francini-Pesenti et al., 2018; Francini-Pesenti et al., 2020). Although these studies were constrained by cross-sectional design, potential referral bias, and reliance on ultrasound for the majority of cases, they position NAFLD and steatohepatitis as prominent, but not fully quantified, systemic features of SBMA that may contribute substantially to morbidity and long-term mortality.

### 4.3. Liver disease in DRPLA and SCAs

In DRPLA, overt primary liver disease has been reported infrequently, but childhood-onset series highlight profound hypoalbuminemia. The largest cohort study reported to date used serial biochemistry and multi-organ histology and observed that serum albumin was lost into organs such as the kidneys, lungs, and gastrointestinal tract, and that atrophin-1-positive, polyQ-containing inclusions were present in multiple peripheral tissues, supporting a systemic vascular or barrier defect rather than isolated hepatic failure (Nagai et al., 2013). Complementary case reports described progressive hematuria, proteinuria and biopsy-supported glomerular disease in DRPLA, indicating that clinically relevant renal involvement can occur as part of the multisystem phenotype (e.g., Morita et al., 2007).

In SCAs, primary liver disease was reported mainly in the context of infantile-onset SCA7. Infantile SCA7 cases with pathologically large ATXN7 expansions consistently present as multisystem disorders: affected infants can develop cardiomyopathy or other structural heart disease, renal dysfunction or failure, hepatomegaly or liver dysfunction, severe failure to thrive, and capillary-leak–type multiorgan failure, with death in early childhood (e.g., Benton et al., 1998; Whitney et al., 2007; Trang et al., 2015; Donis et al., 2015; Gousse et al., 2018; La Spada, 2026; Goswami et al., 2022). By contrast, adult-onset SCA7 and other dominant SCAs are not generally characterized by frank hepatic failure in cohort studies, and most reports of liver involvement are isolated cases rather than systematic findings. Given the high background prevalence of non-alcoholic fatty liver disease and other hepatopathies, targeted hepatology sub-studies within genotype-defined SCA cohorts, incorporating liver enzymes, elastography or MRI-PDFF and biopsy in selected cases, will be required to determine whether polyQ expansions confer any additional hepatic risk beyond lifestyle and metabolic factors.

Next, we discuss cardiovascular manifestations, including autonomic measures and cardiac structure and rhythm.

## 5. Cardiovascular system

Patient-derived data reveal clinically relevant autonomic, conduction, and structural heart abnormalities across several genotypes in polyQ diseases. Here, we summarize information pertaining to HD and SBMA, as well as the polyQ SCAs. Due to a lack of information on DRPLA, this disease has been excluded from this section.

### 5.1. Cardiac autonomic and electrical anomalies in HD

In HD, multiple clinical studies using standardized autonomic testing reported reduced heart rate variability (HRV) and impaired cardiovagal modulation, often detectable even in premanifest or early stages (Sharma et al., 1999; Andrich et al., 2002; Marotta et al., 2021; Terroba-Chambi et al., 2021; Kobal et al., 2022). An earlier investigation documented diminished blood pressure response to handgrip and prolonged pupillary light reflex latency in HD (Den Heijer et al., 1988). Conduction abnormalities, arrhythmic features, and markers of cardiac electrical remodeling are also recognized in HD. In early symptomatic HD, standard ECG studies have reported bradycardia, QRS prolongation, QTc prolongation, and other conduction abnormalities, whereas other ECG and Holter studies have identified repolarization/remodeling abnormalities, arrhythmic events, and altered autonomic regulation (Stephen et al., 2018; Cankar et al., 2018; İşcan and Çetinkaya, 2024). While these data support clinically meaningful cardiac involvement, dedicated prospective cardiology cohorts with systematic echocardiographic characterization of cardiomyopathy are sparse and much of the direct evidence for structural and functional cardiac remodeling emerged from transgenic HD mouse models (Mihm et al., 2007; Mielcarek et al., 2014; Schroeder et al., 2016; Toczek et al., 2016; Critchley et al., 2018; Dridi et al., 2020). Given the burden of cardiovascular mortality in HD and the broader systemic phenotype, including weight loss, metabolic disturbance, and peripheral inflammation, prospective studies incorporating echocardiography, ECG, and HRV into routine HD assessments represent an important unmet need.

### 5.2. Cardiac involvement in SBMA

In SBMA, electrocardiograms from individuals with genetically confirmed SBMA revealed Brugada-type ECG patterns, which may indicate increased arrhythmic susceptibility (Araki et al., 2014). A subsequent study evaluated SBMA patients and controls and found a high prevalence of abnormal ECGs (Brugada-like patterns, early repolarization, and fragmented QRS) together with subtle structural abnormalities and diffuse myocardial fibrosis on cardiovascular MRI, raising concern for increased arrhythmic risk, even though overt systolic dysfunction was uncommon (Steinmetz et al., 2022). Additional, recent data from a cohort study further indicate phenotypic heterogeneity in SBMA and a measurable burden of cardiovascular comorbidities (Roos et al., 2026). Phenotypes observed in this study included hypertension, diabetes mellitus, and cardiac disease, with some cardiac manifestations reported before or around motor symptom onset (Roos et al., 2026). These findings support the inclusion of ECG- and cardiac-based measures as practical peripheral endpoints for longitudinal monitoring and, where appropriate, treatment-response assessment in SBMA. While the absolute risk of lethal cardiac events in SBMA is uncertain, the available data support consideration of baseline and symptom-directed cardiac evaluation; prospective studies are needed to define screening intervals and clinical benefit.

### 5.3. Autonomic dysfunction and HRV in SCAs

The best-characterized cardiovascular manifestation across polyQ diseases comes from studies of autonomic regulation in the SCAs. One cross-sectional HRV analysis examined 22 genetically confirmed patients with SCA1, SCA2, and SCA3 (11, 6, and 5, respectively) alongside matched controls and found that both time- and frequency-domain indices were significantly reduced in the SCA group (Pradhan et al., 2008). The reductions were particularly prominent in parasympathetic-related measures and in overall variability, consistent with cardiac autonomic dysfunction. Sympathetic indices were also diminished, and the low-frequency to high-frequency ratio did not rise, arguing for generalized autonomic failure rather than a simple shift toward sympathetic dominance; important limitations included modest per-genotype sample sizes and the absence of structural cardiac imaging (Pradhan et al., 2008).

Subsequent investigations in SCA2 expanded these observations with multimodal autonomic testing. In a cross-sectional study of symptomatic SCA2 patients and matched controls, HRV analysis was paired with standardized autonomic reflex testing, revealing marked HRV suppression, abnormal blood pressure responses, and evidence of both cardiac sympathetic and parasympathetic dysfunction that correlated with disease duration and ataxia severity (Montes-Brown et al., 2010). A complementary analysis of presymptomatic SCA2 mutation carriers and matched controls noted that HRV anomalies and subtle reflex-test changes can be detected before overt ataxia, supporting cardiac autonomic involvement as an early systemic feature (Montes-Brown et al., 2012).

Evidence for autonomic disturbance also extends to SCA3. In a study of genetically diagnosed SCA3 patients and healthy controls, resting spectral analysis reported tachycardia with reduced overall HRV, consistent with sympathetic–parasympathetic imbalance (Asahina et al., 2010). Additional studies incorporating baroreflex sensitivity testing in SCA1 and SCA2 further support impaired cardiovagal control in these disorders (Tamuli et al., 2019). Altogether, cardiac autonomic abnormalities have been documented in SCA1, SCA2, and SCA3, including presymptomatic SCA2, but their prevalence and clinical consequences across polyQ SCAs remains to be determined.

Cardiovascular phenotypes often co-occur with changes in body composition and frailty, linking autonomic and cardiac physiology to musculoskeletal and metabolic health. In the following section, we overview evidence for bone density, adipose tissue, and overall body composition changes.

## 6. Bone, adipose tissue and body composition

Alterations in bone mineral density and body composition provide another line of evidence that select polyQ diseases exert clinically important effects on peripheral tissues beyond the central nervous system.

### 6.1. BMD and body composition in HD

Evidence that bone and body composition are affected early in HD comes from studies spanning the premanifest and manifest stages. As mentioned already, in premanifest gene carriers, bone mineral density z-scores were significantly lower than in controls, even after adjustment for age, sex, and body size. These differences were not explained by testosterone, cortisol, leptin, or vitamin D levels, supporting an early mutation-associated effect on bone metabolism (Goodman and Barker, 2011). In manifest HD, a body composition study reported reductions in lean mass, fat mass, and bone mineral density compared with controls. Lower lean mass and altered fat distribution predicted subsequent weight loss, implicating progressive sarcopenia and fat loss as key contributors to the cachexia that is commonly observed in HD (Costa de Miranda et al., 2019). Complementary work linked reductions in muscle and bone mass to clinical severity, reinforcing the clinical relevance of these systemic changes (Turner et al., 2020). These collective findings again indicate a broader disturbance of energy balance and endocrine signaling in HD, as already alluded to above. Given the elevated risk of falls and fractures in this disease due to motor and balance impairments, reduced bone mineral density and muscle strength likely contribute meaningfully to morbidity and suggest that early screening and management of osteoporosis and related risk factors is beneficial in this population.

### 6.2. SBMA and other polyQ diseases

In SBMA, hypogonadism and androgen insensitivity would be expected to predispose to osteoporosis and altered body composition. While relatively few dedicated densitometry studies exist, there is strong documentation of increased incidence of bone fractures, and reduced BMD in SBMA patients, associated with decreased bone turnover, and lower vitamin D levels (Kawase et al., 2025). Body composition is altered in SBMA, with progressive muscle loss accompanied by metabolic abnormalities such as insulin resistance and NAFLD; evidence for increased visceral adiposity is more variable across cohorts. (Francini-Pesenti et al., 2018; Francini-Pesenti et al., 2020). In SCAs and DRPLA, bone and adipose tissue have rarely been systematically examined in primary research studies. Nonetheless, chronic immobility, malnutrition, and systemic factors, such as hypoalbuminemia in DRPLA, reviewed above, likely contribute to osteopenia and sarcopenia. Prospective DEXA and body composition studies in these disorders could help distinguish primary polyQ-related effects from secondary consequences of disability.

Alterations in body composition and connective tissues raise the possibility that polyQ expansions impact additional peripheral compartments that are accessible for biopsy and mechanistic study. We therefore turn to skin and other peripheral tissues.

## 7. Skin and other peripheral tissues

Beyond muscle, liver, and cardiac involvement, polyQ diseases also affect skin, fibroblasts, gonads, adipose tissue, and other peripheral organs, offering additional windows into their systemic pathology.

### 7.1. Dermal involvement in HD and DRPLA

Early evidence for cutaneous involvement in HD came from a small but systematic histopathologic analysis of skin biopsies from clinically affected patients. Despite the absence of obvious macroscopic skin changes, biopsies reported hyperkeratosis, epidermal atrophy, subepidermal fibrosis, and increased dermal acid mucopolysaccharides, pointing to subclinical skin pathology (Witkowski et al., 1984). Peripheral fibroblasts were examined as an accessible cellular system. An initial, culture-based study reported that fibroblasts from clinically diagnosed HD patients reached higher maximal cell densities and displayed altered growth characteristics compared with controls, suggesting a genetically determined alteration in cell metabolism or surface properties (Goetz et al., 1975). However, a subsequent study by the same group did not reproduce these differences, finding no significant divergence between HD and control cells across the tested growth and culture parameters (Goetz et al., 1981). More recent work leveraging gene-confirmed, patient-derived fibroblasts and complementary mouse and cellular HD models has expanded onto this peripheral-cell perspective (Gines et al., 2003; Seong et al., 2005; Siddiqui et al., 2012). In these more recent studies, impaired mitochondrial function and altered purine metabolism emerged as recurring features of HD peripheral cells that parallel changes reported in muscle and brain (Tomczyk et al., 2021; Hung et al., 2018).

Skin involvement in DRPLA has been supported by biopsy-based evidence that epidermal cells can harbor expanded polyQ proteins. In two patients, epidermal biopsies revealed multiple clear cells that were immunoreactive with an antibody recognizing expanded polyQ tracts. Immunophenotyping (p63-positive, S-100–negative, cytokeratin 20–negative) was consistent with keratinocyte identity, and ultrastructural analysis indicated decreased tonofilaments, suggesting cytoskeletal disruption (Ohta et al., 2009). These observations indicate that keratinocytes themselves can accumulate expanded polyQ proteins in DRPLA, supporting the broader view that peripheral tissues such as skin can mirror central nervous system aggregation pathology. At the same time, the study's design, a two-patient, qualitative case series without functional skin measurements, positions it as proof-of-principle rather than a basis for population-level estimates of cutaneous involvement.

### 7.2. Gonads, adipose tissue, and other organs in SBMA and HD

In SBMA, endocrine and metabolic features converge on gonadal and adipose phenotypes that are clinically recognizable. Testicular atrophy, spermatogenic failure, and histologic evidence of seminiferous tubular degeneration are common, consistent with partial androgen insensitivity and with direct AR toxicity in gonadal tissue (Dejager et al., 2002; Valera Yepes et al., 2015). Gynecomastia is likewise highly prevalent and can precede neuromuscular symptoms, making it an important clinical clue in men who present with otherwise unexplained endocrine findings (Dejager et al., 2002). These reproductive features often accompany broader metabolic remodeling, aligning with metabolic syndrome and NAFLD (Francini-Pesenti et al., 2018, 2020). In HD, body composition studies mentioned above also described altered fat distribution with changes in subcutaneous and truncal depots alongside data suggesting relative preservation of visceral fat despite overall weight loss (Goodman and Barker, 2011; Costa de Miranda et al., 2019).

Beyond adipose tissue, reports of visceral organ involvement in polyQ disorders are heterogeneous. In DRPLA, renal involvement has been described in case-based clinical reports, and multi-organ peripheral pathology with abnormal protein accumulation has also been reported, but these observations do not establish population-level prevalence (Morita et al., 2007; Nagai et al., 2013). In SCAs, severe, early-onset SCA7 has been associated with multi-organ compromise including renal involvement in isolated reports (Whitney et al., 2007). Collectively, these observations underscore the need for systematic imaging and functional studies in living patients to define the frequency and clinical relevance of pancreatic, renal, and other visceral organ involvement.

Peripheral tissues also intersect with circulating immune and blood-based biomarkers that may capture systemic responses to polyQ expansions. Therefore, in the next section we review immune system alterations and blood-based peripheral pathology.

## 8. Immune system and blood-based peripheral pathology

### 8.1. Inflammatory factors in HD

Immune and blood-based abnormalities are among the most consistently reported extra-neural signals in HD, where circulating cytokines, chemokines, and related innate immune factors have been evaluated as accessible biomarkers. A systematic review and meta-analysis of interleukin-6 (IL-6) in HD concluded that IL-6 is consistently elevated in affected cohorts and may track with disease severity (Eide et al., 2023). Targeted profiling studies expanded this blood-based view, identifying abnormal chemokine signatures in HD plasma and cerebrospinal fluid, including elevations in eotaxin and other chemokines implicated in immune cell trafficking (Wild et al., 2011). Additional work reported increased plasma levels of IL-6, MMP-9, VEGF, and TGF-β1 in HD, supporting the potential utility of these markers for disease monitoring (Chang et al., 2015). In a two-cohort evaluation of plasma innate-immune markers in HD, most tested analytes (including multiple complement-related factors) were not significantly different from controls, while C-reactive protein was decreased in early HD; nonetheless, selected markers correlated with clinical measures, (Silajdžić et al., 2013). Blood-based profiling studies in HD, including predominantly cross-sectional cohorts and some longitudinal observations, support persistent, low-grade systemic inflammatory signaling detectable before clinical onset. However, heterogeneous assay platforms, single-center recruitment, and confounding by intercurrent infection and medications complicate direct comparisons across reports. (Bjorkqvist et al., 2008; Wild et al., 2011; Chang et al., 2015; Valadão et al., 2020; Eide et al., 2023).

Although primarily a CNS measure, microglial activation provides context for the systemic inflammatory picture. A multimodal imaging study reported elevated microglial activation in premanifest HD that correlated with five-year probability of clinical onset, supporting the view that innate immune activation is an early feature of the disease process with implications for circulating inflammatory readouts (Politis et al., 2011).

Taken together, these observations fit with the evolving view that inflammatory signaling in HD is likely not merely epiphenomenal but intersects with disease biology in ways that are increasingly relevant to biomarker strategy and therapeutic design.

### 8.2. Signaling disruptions in SBMA and other polyQ diseases

In SBMA, dedicated immune and blood-based profiling is less developed, but systemic comorbidities with inflammatory components, such as NAFLD, metabolic syndrome, and endocrine disruption provide a biologic context in which low-grade inflammation is plausible and may contribute to multisystem burden (Guber et al., 2017; Francini-Pesenti et al., 2020). In the SCAs and DRPLA, formal immune-focused studies are sparse; nonetheless, chronic illness, malnutrition, and autonomic dysfunction may shape infection risk and inflammatory status in ways that are clinically relevant. For example, recent work in SCA3 indicates a measurable peripheral inflammatory signature and suggests that specific circulating inflammatory markers can track with disease- and genotype-related features: peripheral inflammation measures were associated with genetic and clinical manifestations in SCA3, highlighting the potential utility of targeted inflammatory readouts as scalable peripheral biomarkers when interpreted alongside established clinical scales and CAG-related measures (Zhang et al., 2025). In SCA2, blood cell count-derived peripheral inflammatory indices have been linked to clinical phenotype severity; in a cohort of mutation carriers, peripheral inflammatory indices such as NLR, PLR, SII, and AISI were elevated versus controls, and selected indices correlated with speech, non-ataxia, and cognitive measures (Vázquez-Mojena et al., 2023).

Collectively, the findings from these readouts fit within a broader systemic picture of polyQ diseases. Metabolic stress and energy dysregulation recur across disorders, with convergent evidence for impaired oxidative phosphorylation, insulin resistance, and related endocrine and hepatic phenotypes. Peripheral organ involvement broadly reflects where the disease proteins are normally expressed and function, and these systemic manifestations are likely to feed back onto the central nervous system. In this framework, muscle wasting, endocrine failure, NAFLD, and chronic inflammation may actively influence the trajectory of neurodegeneration rather than simply accompany it.

Based on their accessibility, circulating biomarkers are increasingly used for patient stratification and as pharmacodynamic measures in clinical studies. As we conclude this part of the review focusing on non-nervous system pathology, we next turn our attention to the clinical and translational implications of extra-neural pathology.

## 9. Clinical and translational implications

The evidence reviewed here supports the view that polyQ diseases are multisystem disorders with clinically meaningful peripheral involvement. For clinicians, this has several implications. First, routine care could include systematic screening for endocrine and metabolic complications, particularly in HD and SBMA: oral glucose tolerance tests or HbA1c, lipid profiles, and liver function tests can reveal treatable abnormalities. Second, early assessment of bone health and body composition may help prevent fractures and severe sarcopenia through targeted interventions, including nutritional support, vitamin D and calcium supplementation, and pharmacologic osteoporosis treatment. Third, cardiac evaluation, such as ECG, echocardiography, and HRV analysis, should be considered in SCAs and SBMA, especially given evidence of autonomic dysfunction and repolarization abnormalities. Finally, clinicians may recognize that symptoms such as fatigue, muscle cramps, weight loss, and features of metabolic syndrome could be direct manifestations of the underlying polyQ disease rather than unrelated comorbidities.

From a translational perspective, many of the peripheral measures described, including 31P-MRS muscle parameters, body composition indices, BMD, liver fat fraction, endocrine profiles and inflammatory markers are attractive as quantitative, repeatable biomarkers for longitudinal analyses and clinical trials. They may respond more rapidly to systemic therapies than CNS imaging or clinical scales and could augment or complement existing outcome measures. Targeting peripheral manifestations therapeutically, for example by treating insulin resistance, NAFLD, osteoporosis, and sarcopenia, may present additional beneficial effects on neurological progression, although this remains to be tested directly.

A central unresolved question is whether the peripheral abnormalities catalogued here actively contribute to disease progression and severity in the CNS, or instead represent parallel readouts of a shared genetic mutation. Most available patient data are cross-sectional and correlative; therefore, they cannot separate cause from consequence. Longitudinal, genotype-stratified cohorts in which peripheral measures are tracked alongside validated clinical scales would establish whether peripheral changes precede and predict neurological decline. Tissue-specific and peripherally restricted model systems, including conditional expression and tissue-targeted lowering of the mutant protein, would test directly whether peripheral pathology modifies central outcomes. Additionally, interventional studies that correct a defined peripheral abnormality, such as insulin resistance, NAFLD, or sarcopenia, while monitoring neurological endpoints would provide the most direct test of a contributory role.

If peripheral organs are genuine contributors to CNS disease rather than passive bystanders, therapeutic strategies confined to the brain or spinal cord may prove insufficient. CNS-restricted delivery would leave muscle, hepatic, endocrine, and cardiovascular pathology untreated and would not address the bidirectional brain–periphery signaling that may itself influence neurological progression. This notion argues for considering systemic administration of disease-modifying agents, or combined central and peripheral delivery, particularly for modalities such as antisense oligonucleotides and gene therapies whose biodistribution can be engineered. The optimal balance between central and peripheral targeting will ultimately depend on the relative contribution of each compartment, reinforcing the need for the mechanistic studies outlined above. Moreover, considering the growing evidence that systemic cues influence brain aging and neurodegeneration (Bieri et al., 2023; Chuang et al., 2026; Pluvinage and Wyss-Coray, 2020), it is plausible that intervening in peripheral tissues may also modify endocrine signals that influence polyglutamine disease progression in the brain.

As briefly mentioned above, data from the CNS and the periphery may be highly informative in the development and deployment of biomarkers for polyQ diseases. Various peripheral biomarker classes are being developed that can capture systemic disease burden and/or provide scalable trial endpoints. Across several polyQ disorders, neurofilament light chain (NfL) measured in blood (and, where available, CSF) is emerging as a broadly informative marker that can reflect ongoing neuroaxonal injury and may be useful for tracking progression or treatment response. In SCAs, recent syntheses emphasize NfL's potential as a response biomarker across subtypes, alongside assays that directly quantify disease proteins such as expanded ATXN3 for SCA3 trials (Klockgether et al., 2025). Consistent with this, a systematic review of SCA3 biomarkers highlights NfL and polyQ-ATXN3 as the most frequently reported body-fluid biomarkers across SCA3 stages, while also noting additional candidate biomarker categories (oxidative stress, metabolic markers, and miRNAs) that warrant longitudinal validation (Soto-Piña et al., 2024). In HD, comprehensive biomarker overviews emphasize that blood-based NfL complements other molecular measures (including mutant huntingtin in CSF in therapeutic trials) and may be most powerful when integrated with imaging and functional metrics (Aqel et al., 2025).

For disorders with prominent muscle and metabolic pathology, readily available clinical laboratory measures can serve as practical, organ-relevant biomarkers when interpreted in context. CK and other muscle-associated enzymes are commonly elevated in SBMA and have been used as peripheral measures in observational cohorts, while transaminases and metabolic indices can track hepatic and systemic metabolic burden (e.g., insulin resistance, NAFLD-associated profiles, etc.; Aqel et al., 2025; Francini-Pesenti et al., 2018; Roos et al., 2026). Where autonomic or cardiovascular involvement is suspected, standardized autonomic testing and ECG-based metrics can provide additional peripheral endpoints.

An additional approach to discover stage-associated systemic signals is unbiased serum/plasma profiling. Increasingly, stage-stratified serum proteomics is being used to identify progression-linked molecular pathways and candidate early biomarkers in HD, illustrating how peripheral ‘omics’ can complement targeted markers and may help define clinically relevant systemic biology (Christodoulou et al., 2025). More broadly, reviews of brain–periphery interactions in HD highlight that peripheral mediators and lifestyle interventions can modulate systemic physiology and may influence biomarker baselines, underscoring the need to standardize collection and account for confounders such as activity, diet, and comorbidities (Burtscher et al., 2024).

Several reports in SCA3 emphasize that accessible performance measures can provide pragmatic progression markers when biofluid or imaging biomarkers are costly or impractical. For instance, handgrip strength has been piloted as a potential predictor of disease progression in SCA3 (Chiu et al., 2023), suggesting that standardized peripheral functional measures may serve as scalable trial endpoints. Given heterogeneity across organ systems and disease stages, a practical near-term strategy is to deploy composite biomarker panels that pair (i) biofluid markers (e.g., NfL, disease-protein assays where available), (ii) organ-specific clinical chemistry (e.g., CK, metabolic and liver panels), and (iii) functional measures (e.g., strength, autonomic readouts), with prespecified analytic plans for confounding and missingness (Aqel et al., 2025; Klockgether et al., 2025; Soto-Piña et al., 2024).

Finally, for DRPLA in particular, progress toward disease-modifying trials is constrained by the scarcity of well-powered natural-history cohorts and the limited availability of scalable biomarkers that can be implemented across sites. A priority for the field is therefore to build coordinated, longitudinal registries with standardized clinical assessments and biospecimen collection, and to develop practical endpoints that can be repeated over time (for example, blood-based markers and harmonized functional measures), enabling sensitive tracking of progression and treatment response. This emphasis on natural-history infrastructure and broadly deployable biomarkers aligns with current perspectives on accelerating DRPLA therapeutic development in a globally rare repeat-expansion disorder (Chaudhry et al., 2021).

## 10. Concluding remarks

The evidence reviewed here argues that polyQ diseases are not purely brain disorders but multisystem syndromes in which skeletal muscle, endocrine and metabolic organs, liver, heart, bone, skin, and the immune system are genuine disease targets. Recognizing and treating this peripheral pathology will be essential for improving quality of life, refining prognostication, and designing more sensitive biomarkers.

Despite growing recognition of systemic involvement, major gaps remain in cohort size, longitudinal tracking, and harmonized phenotyping across diseases. These gaps point toward a common agenda: larger, earlier, more systematic multisystem phenotyping across the polyQ disorders, coupled with coordinated autopsy and tissue-banking efforts that allow mechanistic questions to be addressed directly. Systematic, genotype-stratified multisystem studies, and coordinated autopsy programs are needed to define how these peripheral changes arise, interact with the CNS, and respond to emerging therapies.

Note: while we made every attempt to include all relevant publications pertinent to this review article, we may have inadvertently missed some of them. We apologize for such omissions.

## Figures and Tables

**Table 1:** The polyglutamine diseases, their causative proteins, mutations, and symptoms.

Disease	Gene	Protein	Inheritance	Normal polyQ range	Pathogenic polyQ range	Key neurological features	Key systemic / non-neurological features	Key references
Huntington's disease (HD)	HTT	Huntingtin	Autosomal dominant	6-35	36-121 (36-39, reduced penetrance)	Chorea, dystonia, incoordination, cognitive decline, psychiatric symptoms (depression, irritability)	Weight loss and cachexia; skeletal muscle atrophy and bioenergetic defects; metabolic alterations including impaired insulin sensitivity; reduced bone mineral density/osteoporosis risk; peripheral inflammatory cytokine alterations; autonomic dysfunction	The Huntington's Disease Collaborative Research Group, 1993; Andrich et al., 2002; Busse et al., 2008; Costa de Miranda et al., 2019; Eide et al., 2023; Ghosh and Tabrizi, 2018; Goetz et al., 1975; Goodman & Barker, 2011; Harms et al., 1997; Hoffmann et al., 2014; Jenkins et al., 1993; Kobal et al., 2022; Lalić et al., 2008; Lodi et al., 2000; Mochel et al., 2007, Mochel et al., 2010; Politis et al., 2011; Saft et al., 2005; Stüwe et al., 2013; Turner et al., 2007; van der Burg et al., 2009; Witkowski et al., 1984
Spinal and bulbar muscular atrophy (SBMA, Kennedy's disease)	AR	Androgen receptor	X-linked recessive	6–36	38-70	Slowly progressive lower motor neuron weakness and atrophy, bulbar involvement, fasciculations, cramps	Partial androgen insensitivity, insulin resistance, NAFLD, altered body composition, osteoporosis/fracture risk, skeletal muscle pathology, and cardiac conduction abnormalities	Araki et al., 2014; Borgia et al., 2017; Dejager et al., 2002; Francini-Pesenti et al., 2018, 2020; Guber et al., 2017; Kawase et al., 2025; La Spada et al., 1991; La Spada and Taylor, 2003; Lombardi et al., 2019; Nakatsuji et al., 2017; Querin et al., 2016; Roos et al., 2026; Rosenbohm et al., 2018; Steinmetz et al., 2022
Dentatorubral–pallidoluysian atrophy (DRPLA)	ATN1	Atrophin-1	Autosomal dominant	3–38	49-88	Progressive ataxia, choreoathetosis, myoclonus, seizures, cognitive decline; juvenile cases with epilepsy and intellectual deterioration	Malnutrition risk; hypoalbuminemia reported in early-onset cases; multisystem organ polyQ accumulation described in autopsy studies	Koide et al., 1994; Lodi et al., 2000; Morita et al., 2007; Nagai et al., 2013; Naito and Oyanagi, 1982; Nucifora et al., 2003; Ohta et al., 2009
Spinocerebellar ataxia type 1 (SCA1)	ATXN1	Ataxin-1	Autosomal dominant	6–34	39-88	Gait and limb ataxia, dysarthria, dysphagia, oculomotor abnormalities, pyramidal signs, brainstem and cerebellar atrophy	Autonomic dysfunction and impaired insulin sensitivity/secretion reported in small studies	Banfi and Zoghbi, 1994; Lalić et al., 2010; Pradhan et al., 2008; Tamuli et al., 2019
Spinocerebellar ataxia type 2 (SCA2)	ATXN2	Ataxin-2	Autosomal dominant	14-31	32-77 (32–38, reduced penetrance)	Progressive cerebellar ataxia, slow saccades, neuropathy, sometimes parkinsonism	Cardiac autonomic dysfunction detectable in presymptomatic carriers; peripheral inflammatory index elevations (NLR, PLR, SII, AISI)	Montes-Brown et al., 2010, 2012; Pradhan et al., 2008; Rezende Filho et al., 2024; Vázquez-Mojena et al., 2023
Spinocerebellar ataxia type 3 (SCA3, Machado–Joseph disease)	ATXN3	Ataxin-3	Autosomal dominant	12–40	55-86	Mixed ataxia, pyramidal signs, dystonia, parkinsonism, peripheral neuropathy, ophthalmoplegia	Cardiac autonomic dysfunction, reduced HRV/tachycardia, peripheral inflammatory alterations, muscle cramps/neuropathy, and occasional primary myopathic pathology	Asahina et al., 2010; Kawaguchi et al., 1994; Leeuwenberg et al., 2026; Li et al., 2023; Pradhan et al., 2008; Rietveld et al., 2020; Stevanin et al., 1995; Zhang et al., 2025
Spinocerebellar ataxia type 6 (SCA6)	CACNA1A	α1A P/Q-type calcium-channel subunit, and C-terminal transcription factor α1ACT	Autosomal dominant	4–18	19-33	Late-onset, relatively pure cerebellar ataxia with dysarthria and nystagmus	Possible primary myopathic changes reported in isolated cases; systematic evidence remains limited	Du et al., 2013; Rietveld et al., 2020; Sasaki et al., 1998; Zhuchenko et al., 1997
Spinocerebellar ataxia type 7 (SCA7)	ATXN7	Ataxin-7	Autosomal dominant	7-18	38-200 or more	Cerebellar ataxia plus progressive cone–rod dystrophy (vision loss), cognitive decline, and rarely seizures	Infantile-onset cases with very large expansions may show multisystem disease; consistent systemic manifestations in adult-onset disease are less well defined.	Benton et al., 1998; Campos-Romo et al., 2018; David et al., 1997; Donis et al., 2015; Goswami et al., 2022; Gousse et al., 2018; Helmlinger et al., 2004; La Spada, 2026; Trang et al., 2015; Whitney et al., 2007
Spinocerebellar ataxia type 17 (SCA17)	TBP	TATA-binding protein	Autosomal dominant	25–43	45-63	Cerebellar ataxia, cognitive impairment/dementia, psychiatric symptoms, extrapyramidal signs; sometimes HD-like phenotype	Limited systematic evidence for consistent systemic manifestations	Lieberman et al., 2019; Todi et al., 2007; Toyoshima et al., 2005; Zühlke et al., 2001

**Table 2:** Abbreviations used throughout the text.

Abbreviation	Full term
31P-MRS	phosphorus-31 magnetic resonance spectroscopy
AIS	androgen insensitivity syndrome
AISI	aggregate index of systemic inflammation
AR	androgen receptor
ATP	adenosine triphosphate
ATXN3	ataxin-3
ATXN7	ataxin-7
BCAA	branched-chain amino acids
BMD	bone mineral density
BMI	body mass index
C3c and C4	complement components C3c and C4
CACNA1A	calcium voltage-gated channel subunit alpha1 A
CAG	cytosine–adenine–guanine trinucleotide repeat
CBC	complete blood count
CDS	cramp disability scale
CK	creatine kinase
CNS	central nervous system
CRP	C-reactive protein
CSF	cerebrospinal fluid
CT	computed tomography
DEXA	dual-energy X-ray absorptiometry
DRPLA	dentatorubral–pallidoluysian atrophy
ECG	electrocardiogram
EM	electron microscopy
EMG	electromyography
FSH	follicle-stimulating hormone
GH	growth hormone
GI	gastrointestinal
GLP-1	glucagon-like peptide-1
HbA1c	glycated hemoglobin A1c
HD	Huntington's disease
HOMA-IR	homeostatic model assessment of insulin resistance
HRV	heart rate variability
HTT	huntingtin
IGF-1	insulin-like growth factor-1
IL-4	interleukin-4
IL-6	interleukin-6
IL-8	interleukin-8
iPSC	induced pluripotent stem cell
IVGTT	intravenous glucose tolerance test
LH	luteinizing hormone
MCS	muscle cramp scale
MeBT	13C-methionine breath test
miRNAs	microRNAs
MLR	monocyte-to-lymphocyte ratio
MMP-9	matrix metalloproteinase-9
MRE	magnetic resonance elastography
MRI	magnetic resonance imaging
MRI-PDFF	magnetic resonance imaging–proton density fat fraction
MRM	multiple reaction monitoring
MRS	magnetic resonance spectroscopy
MS	mass spectrometry
NAA	*N*-acetylaspartate
NAFLD	non-alcoholic fatty liver disease
NASH	non-alcoholic steatohepatitis
NfL	neurofilament light chain
NLR	neutrophil-to-lymphocyte ratio
NMR	nuclear magnetic resonance
OGTT	oral glucose tolerance test
PCr	phosphocreatine
PCR	polymerase chain reaction
PDFF	proton density fat fraction
PET	positron emission tomography
Pi	inorganic phosphate
PLR	platelet-to-lymphocyte ratio
pNfL	plasma neurofilament light chain
polyQ	polyglutamine
pQCT	peripheral quantitative computed tomography
proBNP	pro–B-type natriuretic peptide
QRS	QRS complex
QTc	corrected QT interval
ROC	receiver operating characteristic
SBMA	spinal and bulbar muscular atrophy
SCA	spinocerebellar ataxia
SCA1	spinocerebellar ataxia type 1
SCA2	spinocerebellar ataxia type 2
SCA3	spinocerebellar ataxia type 3 (Machado–Joseph disease)
SCA6	spinocerebellar ataxia type 6
SCA7	spinocerebellar ataxia type 7
SCA17	spinocerebellar ataxia type 17
SCAs	spinocerebellar ataxias (plural)
SHBG	sex hormone-binding globulin
SGLT2	sodium–glucose co-transporter 2
SII	systemic immune-inflammation index
TGF-β1	transforming growth factor beta-1
TNF-α	tumor necrosis factor alpha
TNS	total neuropathy score
TnI	troponin I
TNSc	total neuropathy score, clinical version
TnT	troponin T
TSPO	translocator protein
VEGF	vascular endothelial growth factor

**Table 3:** List of studies reporting non-CNS effects in the polyglutamine diseases.

Disease	Organ/system	Key study (first author, year)	Cohort size	Stage / phenotype	Assay / modality	Design	Main non-CNS findings
**HD**	Skeletal muscle	Lodi et al., 2000	12 HD (4 premanifest), 2 DRPLA,12 controls	Genetically confirmed HD and DRPLA, adults	31P-MRS (calf muscle at rest and post-exercise)	Cross-sectional, case–control	Reduced PCr/Pi ratio and reduced ATP/(PCr + Pi) at rest; reduction in maximal mitochondrial ATP production in symptomatic and premanifest HD; similar deficit in DRPLA
**HD**	Skeletal muscle	Saft et al., 2005	15 HD, 12 asymptomatic mutation carriers, 43 controls	Genetically confirmed HD, symptomatic and asymptomatic adults	31P-MRS (calf muscle at rest and post-exercise); Surface EMG; Immunohistochemistry	Cross-sectional	Significantly slowed PCr recovery after exercise
**HD**	Skeletal muscle	Ciammola et al., 2006	HD patients and controls (cultured primary skeletal muscle cells)	Genetically confirmed HD	Primary skeletal muscle cell culture; apoptosis assays; immunohistochemistry; differentiation assays	In vitro, case–control	Increased susceptibility to apoptosis, HTT-positive inclusions, and abnormalities in differentiation toward mature myotubes in HD-derived muscle cells
**HD**	Skeletal muscle	Turner et al., 2007	HD patients and controls	Genetically confirmed HD	Muscle mitochondrial respiratory-chain (complex II/III) activity; clinical severity and cognitive measures	Cross-sectional	Skeletal muscle mitochondrial respiratory-chain (complex II/III) activity correlated with clinical disease progression and cognitive impairment in HD
**HD**	Skeletal muscle	Busse et al., 2008	20 HD, 20 controls	Genetically confirmed HD, symptomatic adults	Hand-held dynamometry (isometric muscle strength of 6 lower limb muscle groups)	Cross-sectional	Reduced lower limb muscle strength in HD across all 6 muscle groups and both sides
**HD**	Skeletal muscle	Ciammola et al., 2011	HD patients and controls	Genetically confirmed HD	Exercise testing with metabolic monitoring (anaerobic threshold, lactate production)	Cross-sectional	Reduced anaerobic threshold with increased skeletal muscle lactate production during exertion
**HD**	Bone / body composition	Goodman and Barker, 2011	25 HD (all premanifest), 25 controls	Genetically confirmed premanifest HD	DEXA	Cross-sectional, case–control	Lower BMD in premanifest HD; no significant group differences in BMI or percentage fat
**HD**	Bone / body composition	Costa de Miranda et al., 2019	HD patients (manifest) and controls	Manifest HD	Body composition analysis (lean mass, fat mass, BMD)	Cross-sectional	Lower BMD; generally lower lean body mass and truncal fat; body-composition measures correlated with body weight
**HD**	Glucose–insulin homeostasis	Lalić et al., 2008	29 HD, 22 controls	Mostly normoglycemic, various stages	Glucose tolerance assessment; insulin sensitivity; insulin secretion estimation	Cross-sectional	Peripheral insulin resistance with reduced glucose disposal; impaired early-phase insulin secretion despite normal fasting glucose
**HD**	Liver (hepatic mitochondria)	Stüwe et al., 2013	21 manifest HD, 30 premanifest mutation carriers, 36 controls	Manifest HD (early, unmedicated) and premanifest carriers	MeBT; cumulative percentage dose of 13C recovery at 90 min	Cross-sectional	Reduced hepatic mitochondrial oxidative capacity in manifest HD and near-onset premanifest carriers despite clinically normal liver function; breath test results correlated with functional and cognitive decline
**HD**	Liver (hepatic mitochondria)	Hoffmann et al., 2014	25 premanifest HD mutation carriers	Premanifest HD	MeBT; cumulative percentage dose of 13C recovery at 90 min	Longitudinal	Progressive decline in hepatic mitochondrial function in premanifest HD; significant decay in MeBT results, especially in near-onset carriers; changes correlated with cognitive decline and caudate atrophy
**HD**	Brain	Jenkins et al., 1993	18 HD (16 symptomatic, 2 asymptomatic gene carriers), 12 controls (occipital); 15 HD, 8 controls (basal ganglia)	Mostly mild-to-moderate disability	Localized ^1^H NMR spectroscopy	Cross-sectional	Elevated lactate concentrations in occipital cortex and basal ganglia of HD patients correlating with disease duration. (This is a CNS finding that we include due to its implication in hepatic function.)
**HD**	Brain	Harms et al., 1997	17 symptomatic HD, 4 asymptomatic gene carriers, 19 controls	Mixed severity	Proton magnetic resonance spectroscopy; NAA/choline and lactate/choline ratios in frontal cortex	Cross-sectional	Reduced *N*-acetyl-aspartate/choline ratios and elevated lactate/choline ratios in frontal cortex of HD patients. (This is a CNS finding that we include due to its implication in hepatic function.)
**HD**	Systemic metabolism / body composition	Mochel et al., 2007	15 presymptomatic HD, 10 early, 7 mild, 21 controls	Presymptomatic to mildly affected HD	Multiparametric weight balance evaluation; caloric intake assessment; ^1^H NMR plasma spectroscopy; ion-exchange chromatography for branched-chain amino acids; endocrine and inflammatory markers	Cross-sectional	Early hypermetabolic state in HD: significant weight loss despite higher caloric intake in mutation carriers; reduced plasma BCAA that correlated with weight loss and CAG repeat length; reduced IGF1 levels in HD
**HD**	Systemic inflammation	Eide et al., 2023	469 HD, 206 controls (Meta analysis of 9 studies)	Premanifest and manifest (all stages pooled; stage-stratified analyses)	Plasma interleukin-6 (IL-6) across studies; correlation with clinical measures	Systematic review and meta-analysis (through Oct 2021)	Plasma IL-6 meta-analytically higher in mutation-positive individuals vs controls; increases across disease stages; higher IL-6 associated with worse motor impairment and functional disability
**HD**	Inflammation	Politis et al., 2011	8 premanifest HD, 8 symptomatic HD; normal controls (16 MRI, 8 RAC PET, 8 PK PET)	Premanifest vs symptomatic gene carriers (CAG-matched)	MRI volumetry +11C-PK11195 TSPO PET (microglial activation) +11C-raclopride PET (PK/RC)	Cross-sectional, multimodal imaging	Microglial activation higher in premanifest HD in associative striatum and other regions; higher activation correlated with 5-year probability of clinical onset in premanifest carriers. (This is a CNS finding that we include due to its implication with the Immune System section.)
**HD**	Skin / peripheral cells	Goetz et al., 1981	Blind study: 10 coded pairs (HD vs matched controls)	HD vs controls (skin fibroblast lines)	Primary skin fibroblast culture growth parameters (density, population doublings, colony size)	Blinded, paired replication study	No significant differences between HD and control fibroblasts in growth/density or related culture parameters
**HD**	Immune / inflammatory	Silajdžić et al., 2013	Cohort 1: 27 premanifest HD, 27 stage I/II HD, 25 controls; Cohort 2: 14 premanifest HD, 13 stage II/III HD, 15 controls	Genetically confirmed HD, adults	Inflammatory biomarkers (CRP, complement, apolipoproteins) using Luminex assays, ELISA, and MRM-MS	Cross-sectional	No difference among most biomarkers between HD patients and healthy controls in both cohorts; CRP decreased in early HD; selected markers correlated with clinical measures HD fibroblasts reached higher maximal cell
**HD**	Skin / peripheral cells	Goetz et al., 1975	HD patients and matched controls (skin fibroblast lines)	Clinically diagnosed HD	Fibroblast culture growth parameters	Case–control	HD fibroblasts reached higher maximal cell densities and displayed altered growth characteristics compared with controls (not reproduced in Goetz et al., 1981)
**HD**	Skin	Witkowski et al., 1984	HD patients	Clinically affected HD	Skin biopsy histopathology	Case series	Hyperkeratosis, epidermal atrophy, subepidermal fibrosis, and increased dermal acid mucopolysaccharides despite absence of macroscopic skin changes
**SBMA**	Skeletal muscle	Borgia et al., 2017	19 SBMA, 18 controls	Genetically confirmed SBMA, adult males	Muscle biopsy; MS; EM; mitochondrial markers	Cross-sectional	Increased mitophagy and mitochondrial abnormalities in skeletal muscle
**SBMA**	Bone	Kawase et al., 2025	109 SBMA, 21 controls	Genetically confirmed SBMA, adult males	DEXA; fracture history questionnaire; clinical severity measures; motor functions	Cross-sectional	Progressive BMD decrease; high incidence of bone fractures; low bone turnover and vitamin D deficiency
**SBMA**	Skeletal muscle (biomarkers)	Lombardi et al., 2019	SBMA patients and controls	Genetically confirmed SBMA	Muscle-derived and neuron-derived biomarkers; clinical severity measures	Cross-sectional	Muscle-derived, but not neuron-derived, biomarkers correlated with clinical severity
**SBMA**	Endocrine / androgen signaling	Dejager et al., 2002	22 SBMA, 9 AIS, 12 controls	Genetically confirmed SBMA, adult males	Hormone profile (testosterone, LH, FSH, estradiol, SHBG, prolactin); Clinical endocrinology (BMI, gynecomastia onset, testicular volume, fertility history, semen analysis)	Cross-sectional	Partial androgen insensitivity phenotype: gynecomastia, reduced fertility, altered LH/FSH feedback with AR mutations
**SBMA**	Insulin resistance / metabolic	Nakatsuji et al., 2017	55 SBMA, 483 controls	Genetically confirmed SBMA, adult males, mild-to-moderate severity (mean disease duration ~7 years)	Metabolic panel (fasting glucose/insulin, HOMA-IR, lipids), blood pressure, CT fat area; correlations with ALSFRS-R; muscle/fibroblast insulin signaling proteins	Cross-sectional case–control with within-SBMA severity correlations	Higher insulin resistance (HOMA-IR) in SBMA versus controls. (HOMA-IR correlates with worse motor function.) Reduced insulin receptor pathway protein expression in SBMA muscle/fibroblasts
**SBMA**	Metabolic syndrome & NAFLD	Francini-Pesenti et al., 2018	47 SBMA, 123 controls	Genetically confirmed SBMA, adult Italian males	Clinical exam; Fasting labs (glucose, insulin, lipids, HOMA-IR); Ultrasound for NAFLD	Cross-sectional	High prevalence of metabolic syndrome and NAFLD vs population norms; metabolic features track with disease severity (insulin resistance)
**SBMA**	Liver	Guber et al., 2017	Cohort 1: 22 SBMA; Cohort 2: 14 SBMA, 13 female carriers, 23 controls	Genetically confirmed SBMA	Serum chemistries, ultrasound, MRI-PDFF, liver biopsy subset	Cross-sectional, multi-modal	NAFLD highly enriched in SBMA; more severe steatosis and NASH features on histology; hepatic fat had correlation trend with CAG repeat length and metabolic indices
**SBMA**	Cardiovascular conduction	Araki et al., 2014	144 SBMA	Genetically confirmed SBMA, adult Japanese males	ECG; Histopathology; Immunoblotting; PCR	Cross-sectional, case series	Brugada-like ECG changes and ventricular arrhythmia risk in subset of SBMA patients
**SBMA**	Cardiovascular conduction	Steinmetz et al., 2022	30 SBMA, 11 controls	Genetically confirmed SBMA, adult European males	ECG; Cardiac MRI; Blood biomarkers (CK, CKMB, troponin, testosterone, estradiol)	Cross-sectional	High prevalence of early repolarization/J-wave patterns in SBMA, with additional evidence of broader cardiac involvement on multimodal assessment
**SBMA**	Systemic, non-neural phenotype	Querin et al., 2016	73 SBMA, 60 controls	Genetically confirmed SBMA, adult Italian males	Hormone profile (testosterone, LH, FSH); Metabolic profile (glucose, lipids, CK, etc); DEXA; muscle biopsy; ECG	Cross-sectional cohort	High burden of gynecomastia, osteoporosis, metabolic syndrome, conduction anomalies
**SBMA**	Cardiovascular	Roos et al., 2026	49 SBMA	Genetically confirmed SBMA, symptomatic adult males	Blood biomarkers (CK, TnT, TnI, ProBNP, HbA1c, creatinine, pNfL); Repeat expansion analysis; Immunohistochemistry	Retrospective cross-sectional cohort	High prevalence of cardiovascular comorbidities in SBMA, including diabetes mellitus, hypertension, and cardiac disease; cardiac disease occurred before or at motor onset in half of affected individuals
**DRPLA**	Skeletal muscle metabolism	Lodi et al., 2000	2 DRPLA (1 premanifest), 12 HD (4 premanifest), 12 controls	Genetically confirmed DRPLA and HD, adults	31P-MRS of skeletal muscle	Cross-sectional case series	Markedly reduced maximal mitochondrial ATP production even in presymptomatic DRPLA, paralleling HD findings
**DRPLA**	Protein leakage / albumin	Nagai et al., 2013	9 DRPLA (childhood-onset)	Genetically confirmed DRPLA, childhood-onset Japanese patients	Scintigraphy with 99mTc-labeled serum albumin; Histopathological analysis	Retrospective case series	Recurrent hypoalbuminemia with leakage of albumin into multiple organs; vascular leakage and enteric loss suggested
**DRPLA**	Skin	Ohta et al., 2009	2 DRPLA	Genetically confirmed DRPLA, adults	Skin biopsy with immunohistochemistry for atrophin-1	Case series	Atrophin-1 aggregates in epidermal keratinocytes
**SCAs (1/2/3)**	Cardiovascular autonomic	Pradhan et al., 2008	22 SCA (11 SCA1, 6 SCA2, 5 SCA3), 22 controls	Genetically confirmed SCA, adults	HRV	Cross-sectional	Reduced overall HRV, reduced parasympathetic indices
**SCA2**	Cardiovascular autonomic	Montes-Brown et al., 2010	97 SCA2, 97 controls	Symptomatic SCA2	HRV, ECG, autonomic tests	Cross-sectional	Pronounced reduction in HRV indices; abnormal autonomic test battery; dysautonomia correlated with clinical severity
**SCA2**	Presymptomatic autonomic	Montes-Brown et al., 2012	48 presymptomatic SCA2, 48 controls	Genetically confirmed SCA2, presymptomatic adults	HRV at rest and during Valsalva, standing, deep breathing	Cross-sectional	Autonomic abnormalities detectable before overt ataxia
**SCA2**	Immune / inflammatory	Vázquez-Mojena et al., 2023	39 SCA2, 39 controls	Genetically confirmed SCA2	CBC-derived inflammatory indices; clinical scales (ataxia/non-ataxia/cognitive)	Cross-sectional	Elevated peripheral inflammatory markers in SCA2, including increased NLR, PLR, SII, and AISI vs controls; platelet count, CRP, C3c, and C4 also increased. PLR, SII, and AISI elevated also in the preclinical subgroup
**SCA3**	Cardiovascular autonomic	Asahina et al., 2010	10 SCA3, 10 controls	Genetically confirmed SCA3, symptomatic adults (mean disease duration ~11.5 years)	HRV spectral analysis; Head-up tilt test	Cross-sectional, case–control	Reduced high-frequency HRV and reduced low-frequency power
**SCA3**	Immune / inflammatory	Zhang et al., 2025	101 SCA3, 101 controls	Genetically confirmed SCA3	CBC-derived inflammatory indices and CRP; clinical/genetic correlates; ROC	Cross-sectional	Higher leukocytes, neutrophils, monocytes, platelets, NLR, MLR, SII, AISI, and CRP in SCA3 patients; age influenced several inflammatory markers, and expanded-allele CAG repeat length associated with MLR and AISI
**SCA3**	Neuromuscular / autonomic	Leeuwenberg et al., 2026	40 SCA3 (30 ataxic, 10 pre-ataxic), 16 controls	Genetically confirmed SCA3	Patient-reported questionnaires (MCS, CDS, TNSc, 16-item autonomic symptom inventory)	Prospective observational cohort	Weekly muscle cramps in pre-ataxic and ataxic carriers, neuropathic pain, and autonomic symptoms; neuropathy severity increased also in pre-ataxic carriers and correlated with worse function and ataxia severity
**HD / SBMA / DRPLA / SCAs**	Immune / inflammatory	Bjorkqvist et al., 2008; Wild et al., 2011; Chang et al., 2015; Eide et al., 2023; Rosenbohm et al., 2018	From dozens to hundreds per study	Genetically confirmed premanifest and manifest	Plasma cytokines, flow cytometry	Cross-sectional / longitudinal	Persistent low-grade systemic inflammation, altered monocyte and T-cell phenotypes; sometimes correlated with clinical scores

## Data Availability

No data was used for the research described in the article.
